# Decompensated Hypothyroidism in a Patient With Cirrhosis: A Rare Presentation of Severe Hypothyroidism With Hypertension

**DOI:** 10.1016/j.aed.2025.04.002

**Published:** 2025-04-17

**Authors:** Kareem Malas, Grant Herrington, Nam Pham, Adan Mora

**Affiliations:** 1 Department of Internal Medicine, University of Texas-Southwestern, Dallas, Texas; 2 Department of Internal Medicine, Parkland Health, Dallas, Texas

**Keywords:** decompensated hypothyroidism, myxedema coma, cirrhosis, hepatic encephalopathy, hypertension

## Abstract

**Background/Objective:**

Decompensated hypothyroidism (DH) is a rare endocrine emergency with a high mortality rate, particularly challenging to distinguish from hepatic encephalopathy (HE) in patients with cirrhosis due to overlapping symptoms.

**Case Report:**

We present a case of a 79-year-old woman with advanced cirrhosis and a history of multiple HE-related admissions, who exhibited altered mental status, hypothermia, bradycardia, and unusually high blood pressure. Laboratory findings revealed a severely elevated thyroid-stimulating hormone level (335.10 µUI/mL; reference range, 0.40-4.50 µUI/mL) and low free thyroxine level (0.2 ng/dL; reference range, 0.8-1.8 ng/dL), confirming DH. Adrenal insufficiency was ruled out. Intravenous levothyroxine was administered with a dose adjustment over 4 days, leading to significant clinical improvement by hospital day 1 and recovery to baseline by day 4.

**Discussion:**

This case highlights the complexity of diagnosing and managing DH in the context of cirrhosis. Notably, the pronounced hypertension observed may reflect compensatory systemic vasoconstriction in response to a low cardiac output state.

**Conclusion:**

The complexity and severity of concomitant DH and HE underscore the necessity of prompt recognition and tailored treatment to prevent further complications.

**Highlights**

• Decompensated hypothyroidism (DH) can mimic or worsen hepatic encephalopathy (HE), complicating diagnosis in cirrhosis
• Hypothyroidism can elevate ammonia, worsening HE in patients with cirrhosis
• Thyroid function should be re-evaluated in patients with cirrhosis with bradycardia and hypothermia
• DH-induced low cardiac output may cause paradoxical, compensatory hypertension
• Age and comorbidities should be considered when choosing intravenous thyroid replacement strategy

**Clinical Relevance**

The case highlights the diagnostic challenges of decompensated hypothyroidism (DH) in cirrhosis, where symptoms can overlap. This report emphasizes the need for thyroid function evaluation in encephalopathy and importance of individualized thyroid hormone replacement strategies. Awareness of concomitant DH in cirrhosis enables early recognition and treatment of thyroid dysfunction, improving outcomes.

## Introduction

Decompensated hypothyroidism (DH), formerly known as myxedema coma, is a rare endocrine emergency with a 30% to 60% mortality rate. The liver plays an important role in thyroid hormone metabolism with the production of thyroid binding proteins and conversion of thyroid hormones. Differentiating DH from hepatic encephalopathy (HE) can be difficult due to overlapping symptoms.^1^ Manifestations of DH can mask those of decompensated cirrhosis and vice versa. Both share and have opposing pathophysiology and clinical consequences (Fig.). Concomitant presentation offers unique clinical challenges. We report a rare case of DH in a patient with cirrhosis requiring intensive medical care.

**Fig:**
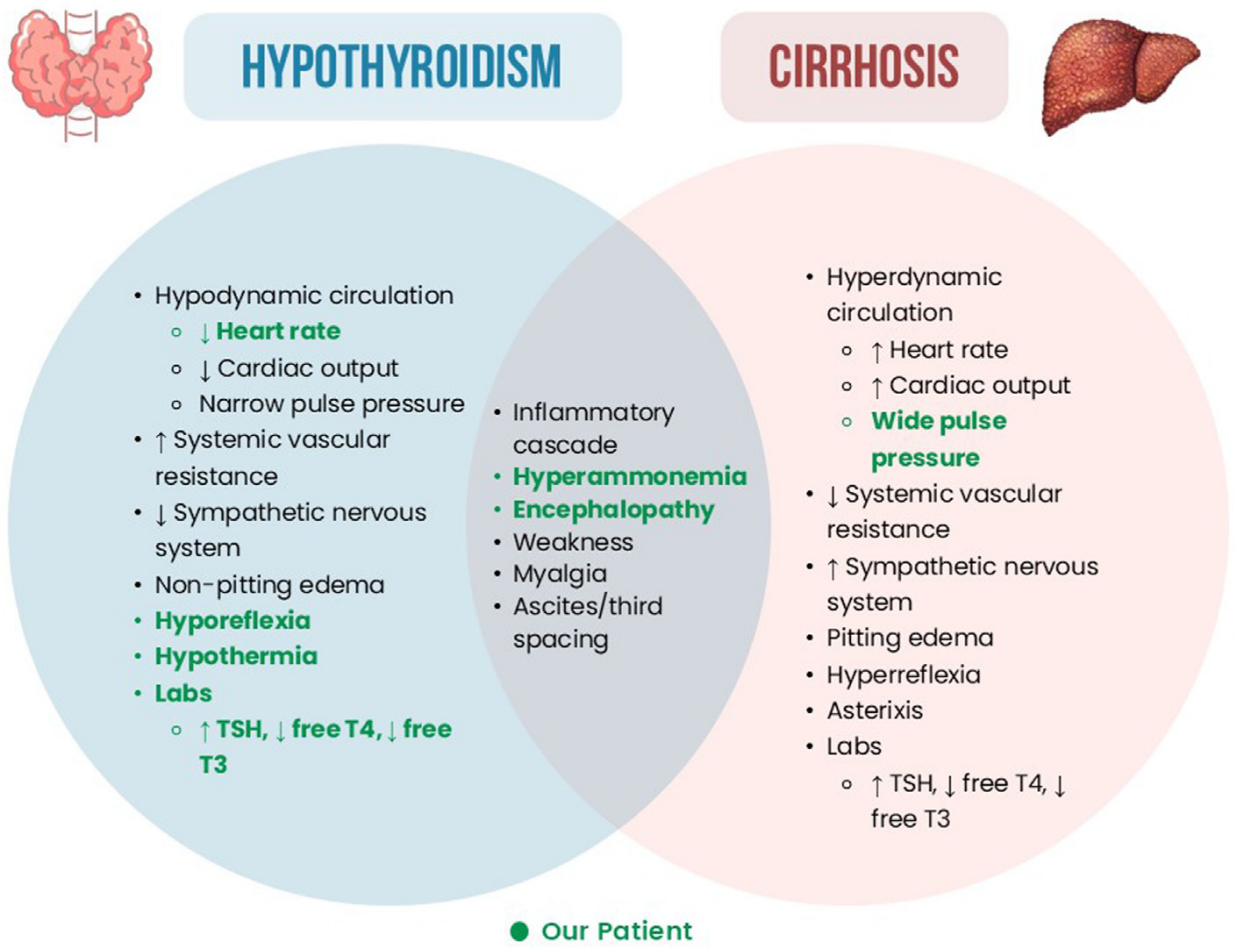
Venn diagram comparing notable clinical findings in hypothyroidism and cirrhosis. The overlapping section represents shared clinical features, whereas unique findings for each condition are shown in the respective nonoverlapping areas. Findings designated in green were observed in our patient case. *T4* = thyroxine; *TSH* = thyroid-stimulating hormone; *T3* = triiodothyronine.

## Case Report

A 79-year-old woman with a past medical history of hypertension (HTN), chronic kidney disease stage V, and advanced metabolic dysfunction–associated steatotic liver disease complicated by esophageal varices, recurrent ascites requiring weekly paracentesis, and multiple admissions for HE presented with 3 days of progressively worsening mental status and cold intolerance. Of note, she was hospitalized 2 months prior for HE and had been compliant with her home lactulose regimen.

On arrival, she was hypothermic (94.3 °F), hypertensive (178/65 mm Hg), and bradycardic (59 bpm). The respiratory rate and oxygen saturation were normal on room air. Pertinent examination findings included the following: (1) ill-appearing and frail, (2) lethargic, (3) moaned to questions, (4) distended but soft abdomen with wincing to palpation, (5) cool extremities, (6) hyporeflexia, (7) absent asterixis, and (8) alert and oriented ×0. Laboratory results revealed a severely elevated thyroid-stimulating hormone level (335.10 µUI/mL; reference [ref.] range, 0.40-4.50 µUI/mL; 2 months prior, 6.15 µUI/mL) and low free thyroxine level (0.2 ng/dL; ref. range, 0.8-1.8 ng/dL; 2 months prior, 1.5 ng/dL). Subsequently, an adrenocorticotropic hormone stimulation test was performed with a baseline cortisol level of 13.2 µg/dL and 60-minute cortisol level of 18.9 µg/dL, ruling out adrenal insufficiency. Other initial studies included the following: (1) aspartate aminotransferase level, 82 units/L (ref. range, 10-35 units/L); (2) alanine aminotransferase level, 21 units/L (ref. range, 10-35 units/L); (3) total bilirubin level, 2.2 mg/dL (ref. range, 0.2-1.3 mg/dL), (4) international normalized ratio, 1.8 (ref. range, 0.9-1.3); and (5) ammonia level, 96 µmol/L (2 months prior, 254 µmol/L; ref. range, 13-40 µmol/L).

Given her advanced age and severe comorbidities, intravenous (IV) thyroid replacement dosing was split over 3 days. Her thyroid replacement consisted of the following: (1) hospital day (HD) #0, IV levothyroxine 150 µg; (2) HD #1, IV levothyroxine 80 µg; (3) HD #2, IV levothyroxine 40 µg + 88 µg orally; (4) HD #3, IV levothyroxine 50 µg; and (5) HD #4, transitioned to stable dose of levothyroxine 88 µg orally daily. The free thyroxine level was trended daily and normalized by HD #1 and was 1.1 ng/dL on HD #4. She demonstrated robust improvement by HD #1 marked by improved mentation and resolution of hypothermia and bradycardia. By HD #4, she recovered to clinical baseline with a temperature of 98.1 °F, blood pressure of 142/63 mm Hg, and heart rate of 78 bpm. A summary of the hospital course with information including thyroid function and clinical status can be visualized in the Table.

**Table:** Summary of Hospital Course by Hospital Day, Thyroid-Stimulating Hormone Level (µIU/mL; Reference Range, 0.40-4.50 µIU/mL), Free Thyroxine Level (ng/dL; Reference Range, 0.8-1.8 ng/dL), Levothyroxine Dosing, and Clinical Status

HD #	TSH (µIU/mL)	Free T4 (ng/dL)	Levothyroxine	Clinical status
−2 mo	6.15	1.5	None	HE admission
0	335	0.2	IV 150 µg	Critically ill
1	…	1.2	IV 80 µg	Robust improvement
2	…	1.0	IV 40 µg + 88 µg orally	…
3	…	1.2	IV 50 µg	…
4	…	1.1	88 µg orally	Recovery to baseline

Abbreviations: HD = hospital day; HE, hepatic encephalopathy; IV = intravenous; T4 = thyroxine; TSH = thyroid-stimulating hormone.

HD # “−2 mo” refers to a hospitalization for HE 2 months prior to this case.

## Discussion

Our patient had signs/symptoms of sudden-onset DH with atypical features in a background of HE and HTN. Although diastolic HTN has been described in hypothyroidism, this patient had pronounced systolic HTN with a wide pulse pressure over 100 bpm. Cirrhosis is a state of hyperdynamic circulation with increased cardiac output and reduced systemic vascular resistance. We propose that without evidence of adrenal insufficiency, peripheral vascular HTN was compensatory for DH-induced low cardiac output, which later may have progressed to shock if untreated. Although hypothermia is uncommon in HE, patients with decompensated cirrhosis are at increased risk of sepsis, a potential cause of hypothermia. However, a broad infectious workup was negative on presentation. In the absence of infection, the hypothermia was attributed to DH via impaired metabolism and reduced heat production. Hypothyroidism is theorized to cause increased ammonia due to decreased protein synthesis, increased protein catabolism, decreased intestinal motility that promotes bacterial ammonia production, and decreased glutamine synthase activity.^2^ Rare cases such as this have been reported with hyperammonemia that did not improve with standard HE treatment and were found to have DH, which responded to levothyroxine treatment.^3^, ^4^, ^5^, ^6^ A low free triiodothyronine level has been associated with systemic inflammation, acute-on-chronic liver failure, and mortality, whereas a low thyroxine level has been associated with the development of advanced HE.^7^^,^^8^ Free thyroid hormone reduction is often indicative of thyroid compensation to a nonthyroidal illness, that is, euthyroid sick syndrome. She was not given hydrocortisone because the baseline cortisol was adequate and adrenocorticotropic hormone stimulation test was robust. IV levothyroxine was administered due to suspected low bioavailability due to gut edema observed in DH, and lower doses were chosen given arrhythmia risk related to age and other risk factors. Despite a very recent thyroid evaluation during her prior admission, thyroid-stimulating hormone was tested given the peculiar findings of hypothermia, bradycardia, and cold intolerance. This case also served as a testament to avoid anchoring bias that would lead to the assumption that her presentation was driven by HE similar to prior hospitalizations. It was not clear why the patient developed such drastic hypothyroidism despite demonstrating stable subclinical hypothyroidism just 2 months prior. There were no evident triggers such as adverse medication effects or infection. However, there have been reports of similar presentations of rapid progression to DH in elderly individuals with high antithyroid antibody titers.^9^ Antithyroid antibody was not evaluated in this patient, and thus, it cannot be determined whether this mechanism contributed to her presentation.

## Conclusion

This case highlights the importance of recognizing DH as a potential contributor to altered mental status in patients with cirrhosis. The interplay between DH and HE presents unique diagnostic and management challenges, requiring a high index of suspicion and thorough evaluation. Our case underscores the need for repeated thyroid function testing in critically ill patients, even those with recent thyroid assessments, when atypical findings such as hypothermia, bradycardia, and cold intolerance are present. Furthermore, the paradoxical hemodynamic presentation of systolic HTN observed in our patient raises questions about compensatory mechanisms in cirrhosis that warrant further exploration. Tailoring the approach to IV thyroid replacement in these critical scenarios, with heavy consideration of the patient’s advanced age and comorbidities, offers a delicate perspective on the management of DH in patients with cirrhosis. By presenting this case, we aim to enhance awareness of DH in patients with advanced liver disease, promote early recognition, and optimize management strategies to improve patient outcomes.

## Disclosure

The authors have no conflicts of interest to disclose.
